# Supplementary material for: Three eruptions at the Fagradalsfjall Volcano in Iceland show rapid and predictable microbial community establishment
Source: Commun Biol. 2025 Nov 24;8:1657. doi: 10.1038/s42003-025-09044-1 (PMC12644735; doi:10.1038/s42003-025-09044-1)
Supplement: Supplementary file 2 — Supplementary Information [file 42003_2025_9044_MOESM2_ESM.pdf]

Supplementary Information for

Three Eruptions at the Fagradalsfjall Volcano in Iceland Show Rapid and Predictable Microbial Community Establishment

Nathan Hadland[1], Christopher W. Hamilton[1], Snædís Björnsdóttir[3], Solange Duhamel[1,2*]
[1]Lunar and Planetary Laboratory, University of Arizona, Tucson, AZ USA
[2]Molecular and Cellular Biology, University of Arizona, Tucson, AZ USA
[3]Life and Environmental Science, University of Iceland, Reykjavik, Iceland.

Corresponding author: *Solange Duhamel.
Email: duhamel@arizona.edu

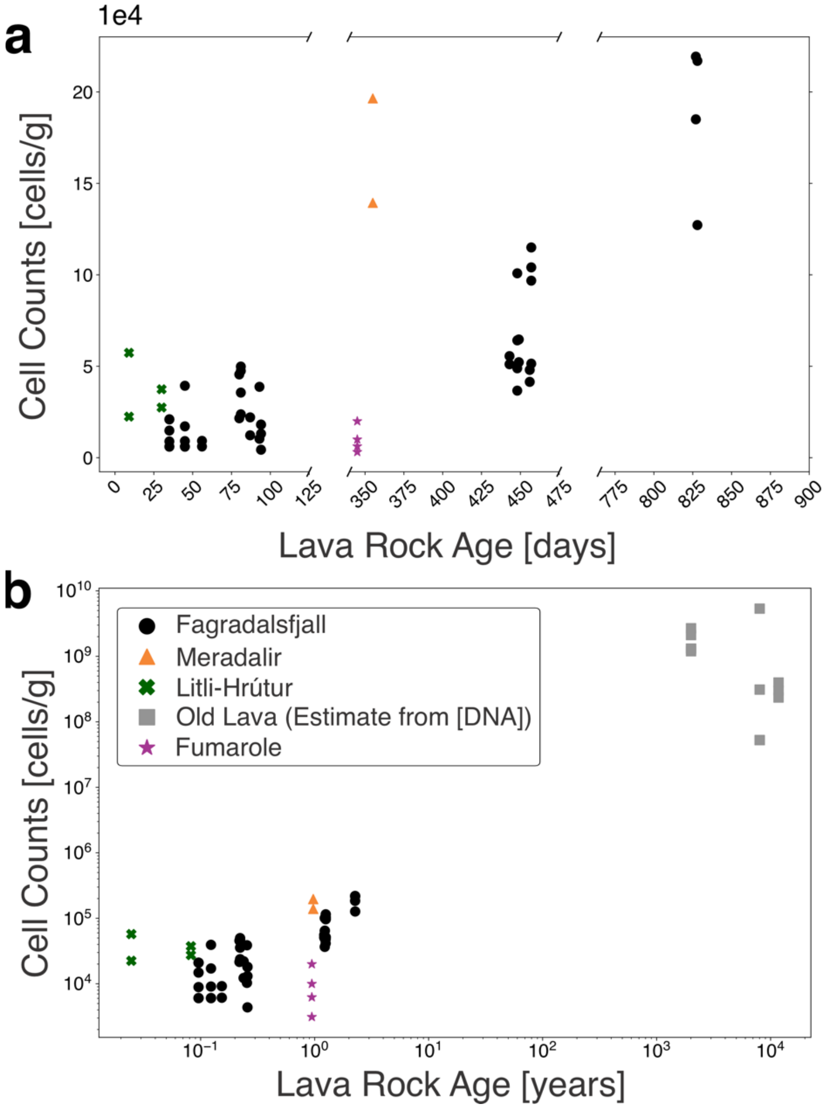

Supplementary Fig. 1. Abundances of cells detected by fluorescent microscopy in lava samples. (a) Cell counts across multiple eruptions (cells/g dry weight of lava). Samples had duplicate slides prepared and both points are plotted. (b) Cell counts including estimates of cell abundances in old lava. Background fluorescence made microscopy of old lava unreliable, so DNA concentrations were converted to cell abundances.

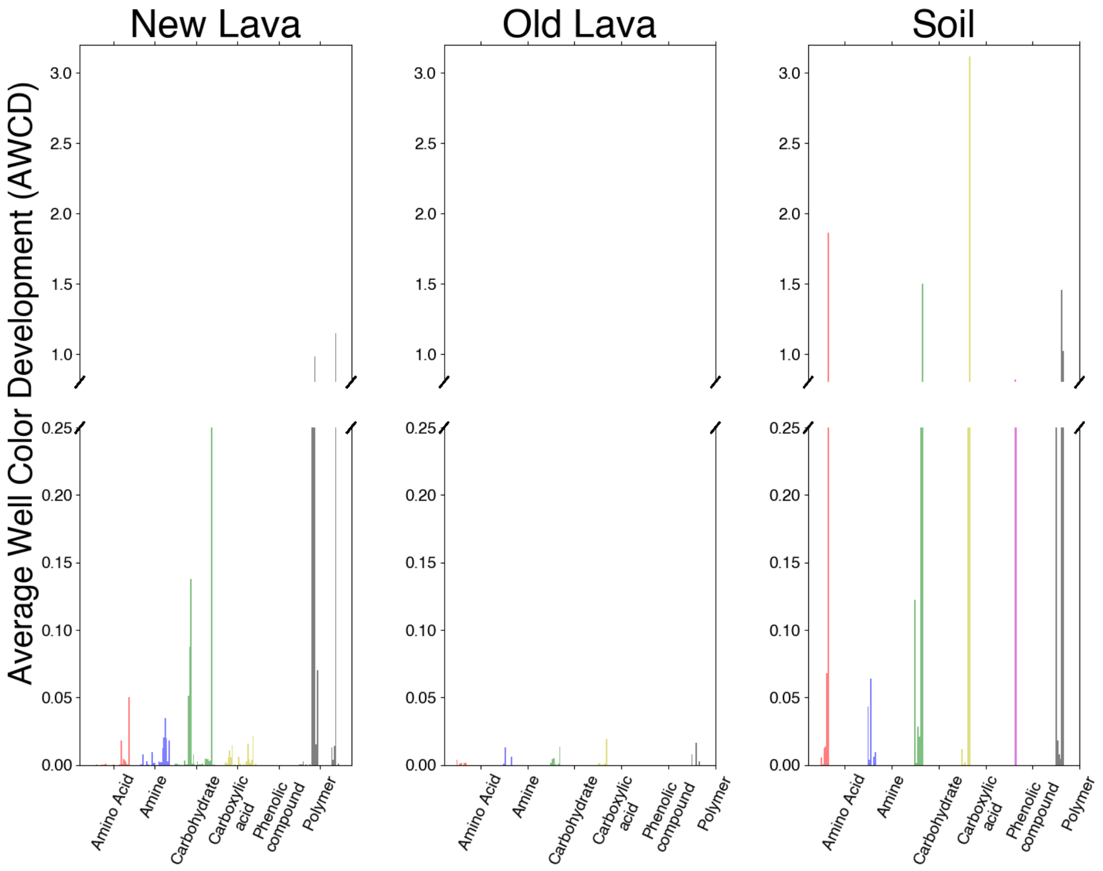

Supplementary Fig. 2. Average well color development for substrate utilization assays. Organic compounds are grouped according to type and plots are separated according to sample type. The different bars in each carbon compound category represent individual samples. Only samples <100 days were subjected to the substrate utilization assay for new lava and are organized from youngest to oldest.

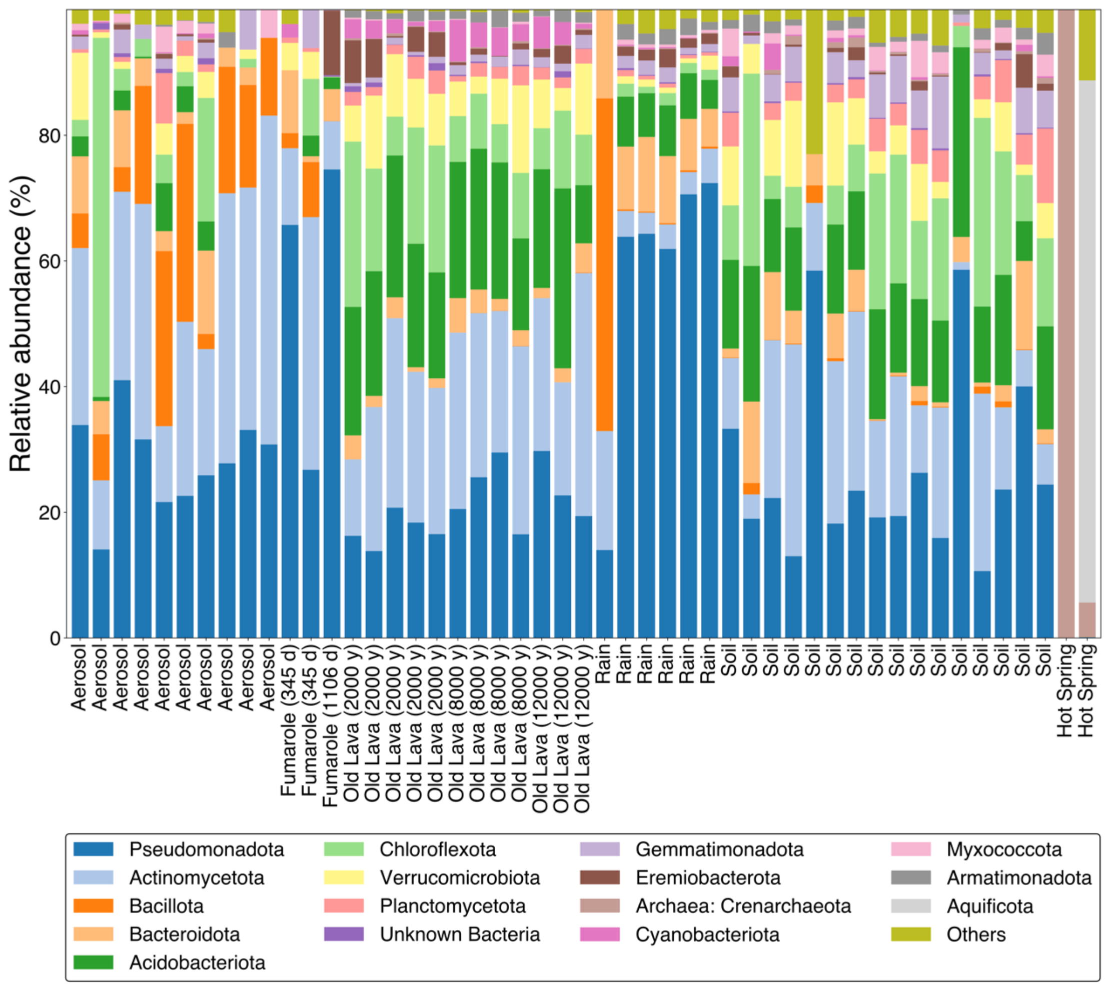

Supplementary Fig. 3 Phyla level bar plots for fumaroles, old lava, and source environments. Fumarole and old lava ages are added in days and years respectively.

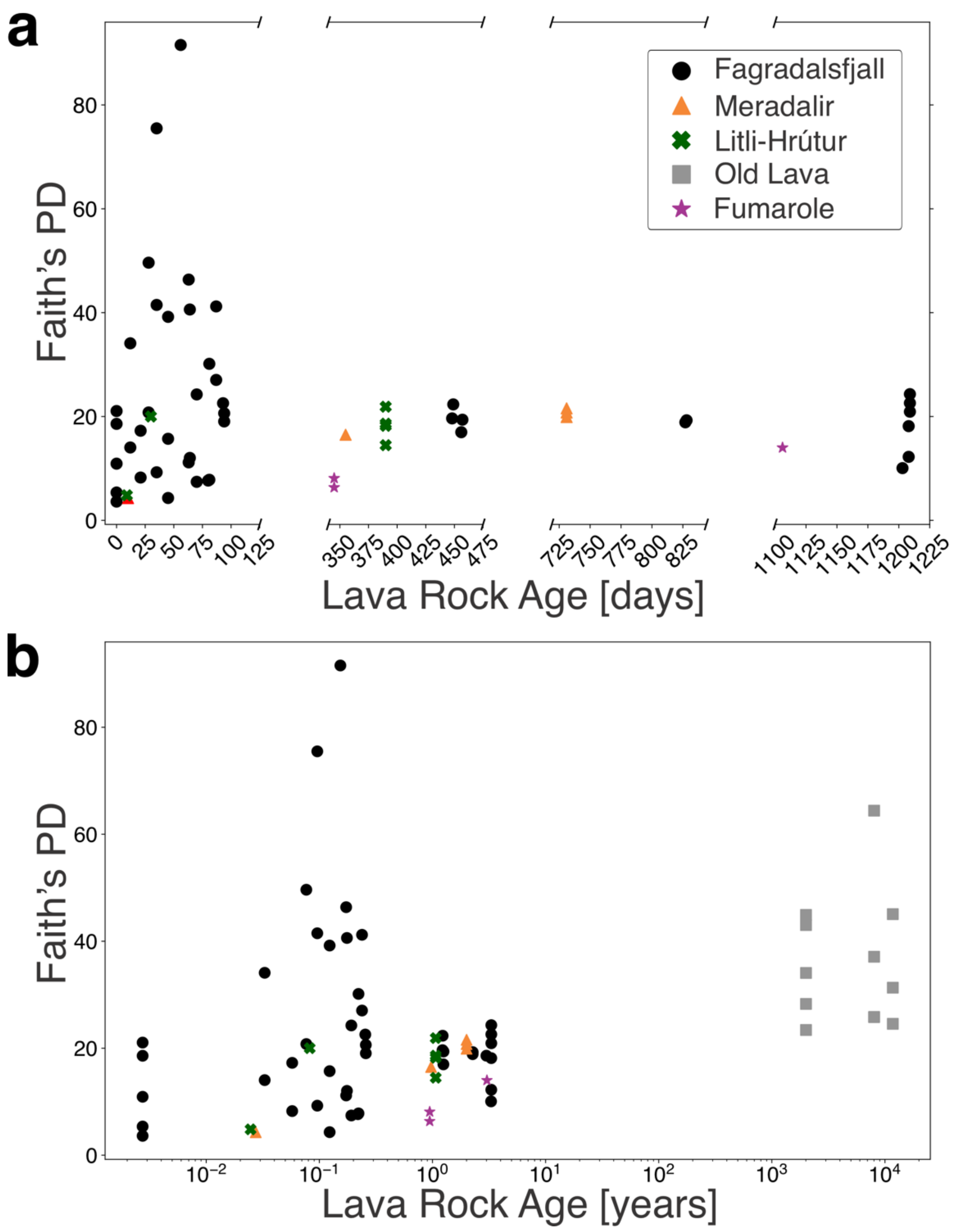

Supplementary Fig. 4. Faith's phylogenetic diversity (PD) vs lava rock age. Data is separated with different markers for the three eruptions for (a) the new lava on a broken axis and (b) with the old lava on a logarithmic scale. The age for samples collected on the first day were set to 1 for visualization on the logarithmic scale.

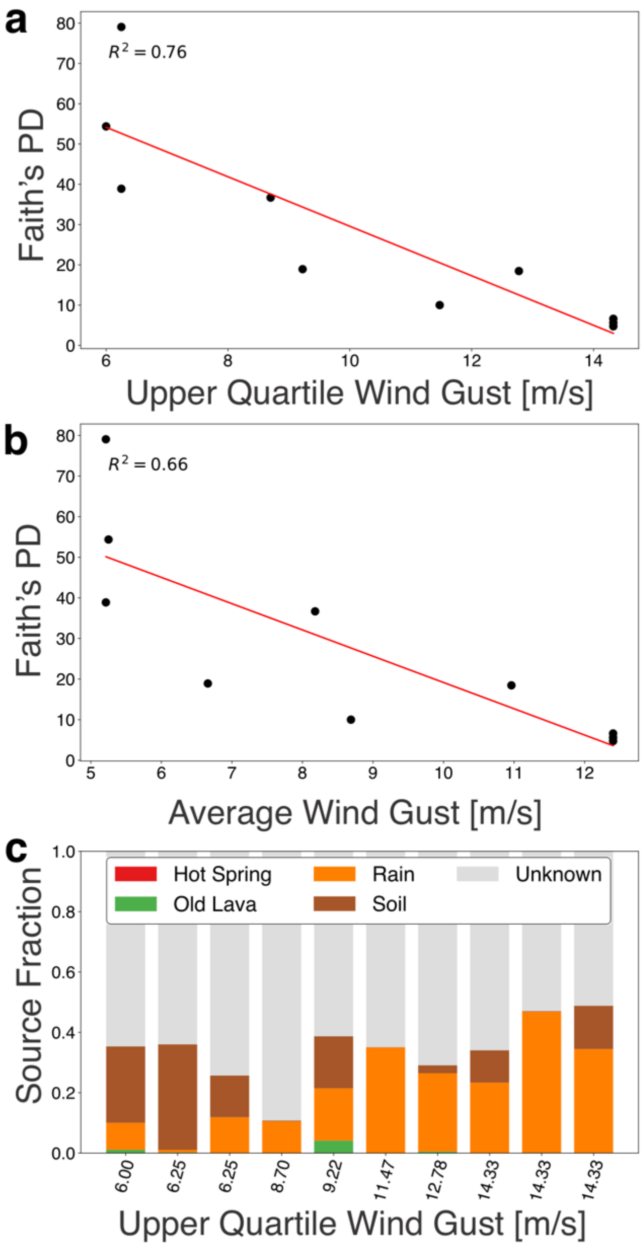

Supplementary Fig. 5. Faith's phylogenetic diversity (PD) for bioaerosols plotted against weather variables (average and upper and lower quartiles) for the 24-hour sampling period. (a) Upper quartile and (b) average wind gust speeds (maximum in 3 seconds) had the highest R^2 association with Faith's PD of these variables. (c) To untangle the potential coupling between ground-based sources and aerosols, the SourceTracker2 analysis was applied using the bioaerosols as the sink. A large proportion of the sequences within the bioaerosol samples were determined to be from an unknown source. However, the proportion of soil was generally lower with higher wind gust speeds. Combined with the lower Faith's PD, these results suggest that lower wind speeds may have caused a higher contribution of local material, such as the soil, to collect on the aerosol filters (resulting in higher Faith's PD) which were mounted 1.5 m above the ground. Alternatively, the higher wind speeds could have carried species well adapted to environmental extremes, such as the atmosphere, from further away. These air parcels would be more diffuse and less diverse.

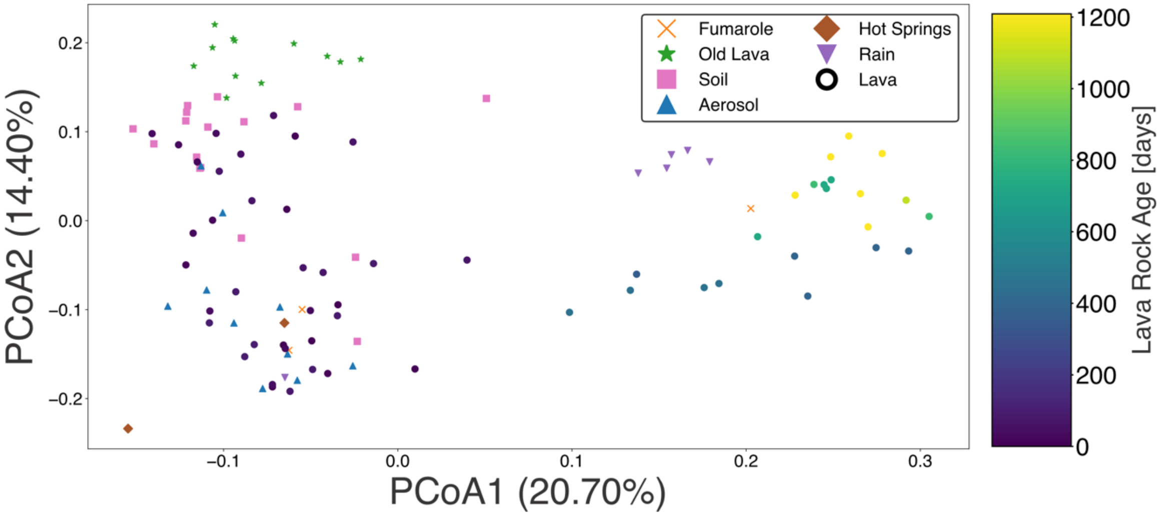

Supplementary Fig. 6. Qualitative source tracking using Principal Coordinate Analysis (PCoA) of the weighted Unifrac metric to show the association of the lava with the different sources. Lava rock samples are colored with respect to age whereas source environments have independent colors and symbols. The aerosols and soil clustered closely with the youngest lava while the rain became more closely associated with the 2–3-year-old lava. Each source clustered independently, though there was some overlap between the aerosols and soil. Pairwise permanova tests for weighted Unifrac showed statistically significant differences between the different sample types (data not shown). While there are potential associations between the sources depending on weather conditions (Supplementary Fig. 4), we chose to continue with the broader categorizations of source because they had distinct enough fingerprints.

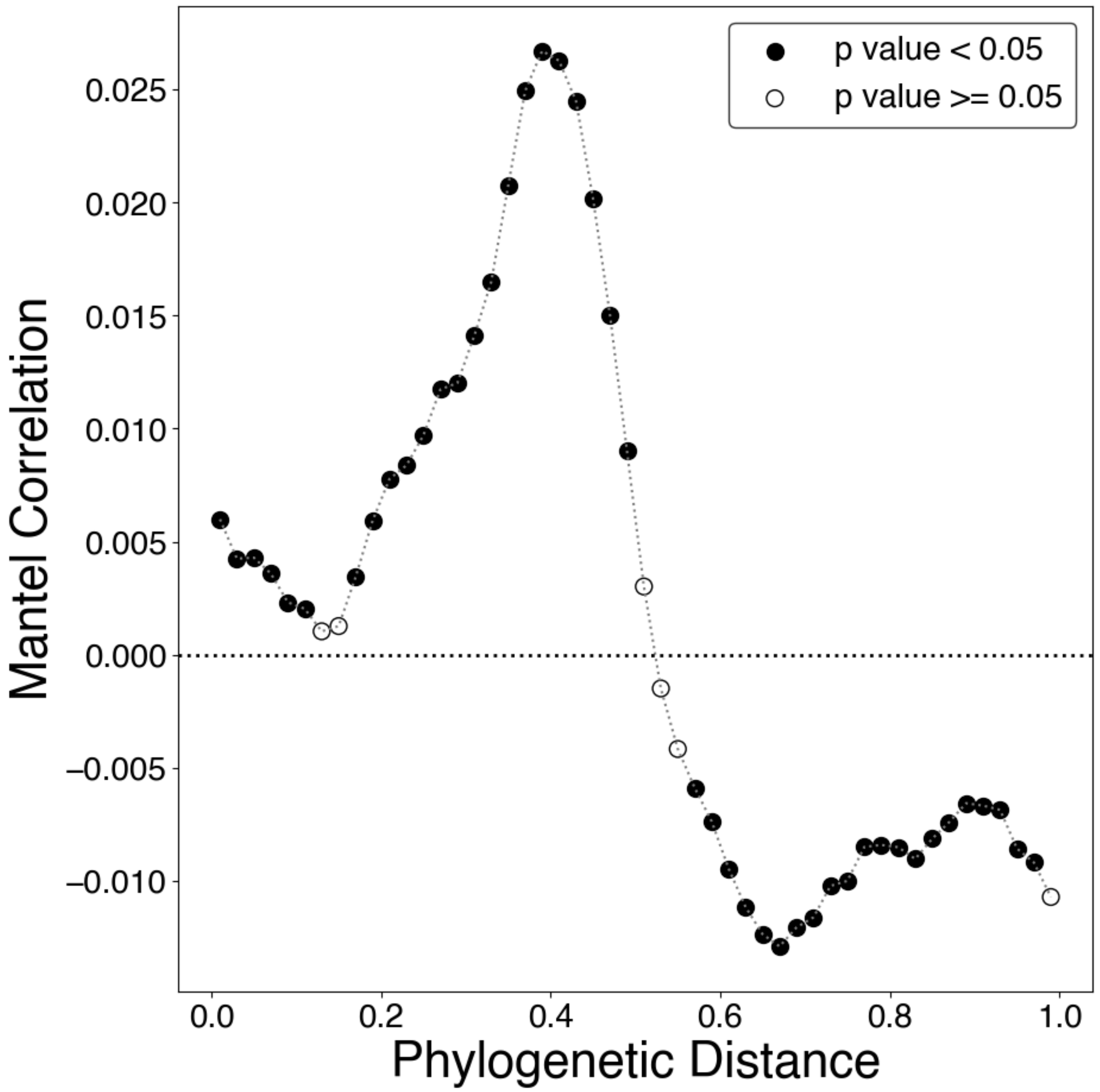

Supplementary Fig. 7. **Mantel correlogram for phylogenetic null modeling.** Pearson correlation resulting from Mantel correlogram (100,000 permutations) between lava rock age—which we consider a proxy for environmental exposure—and phylogenetic distances. Significantly positive mantel correlations (solid circles) indicate phylogenetic signal in ecological niches.

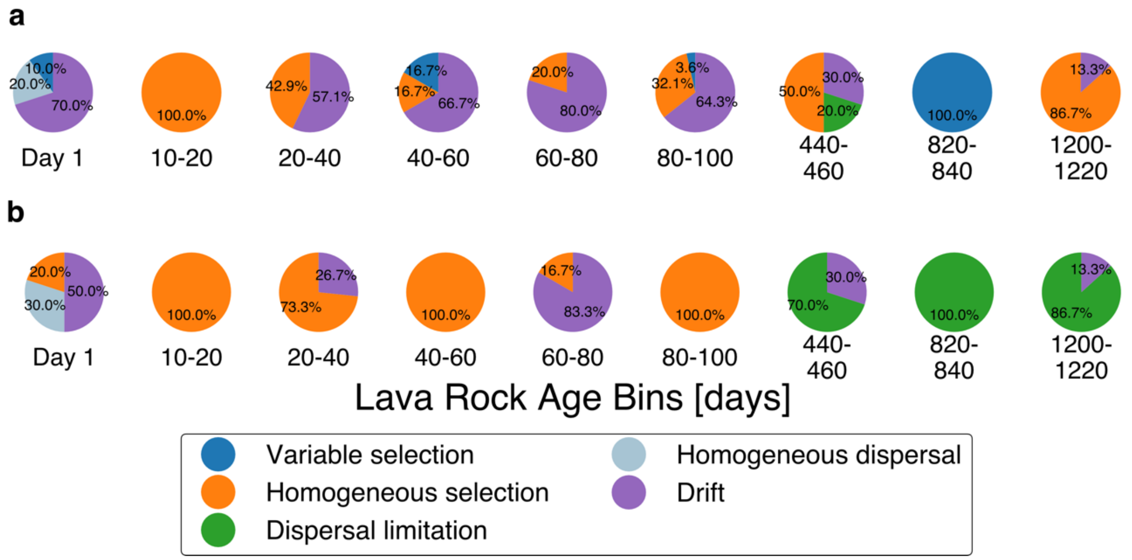

Supplementary Fig. 8. **Processes of microbial community assembly.** (a) Processes of microbial community assembly for Fagradalsfjall samples using the beta diversity component of NTI (βNTI) to partition deterministic processes where |βNTI|>2**,** and the Raup Crick (RC) diversity metric to partition stochastic processes where |βNTI|<2. (b) Leave one out analysis for Site 3 for βNRI, which was only sampled in the first six months because it was overprinted. While there is a slight increase in the contribution of drift, site level effects were determined not to impact the results significantly.

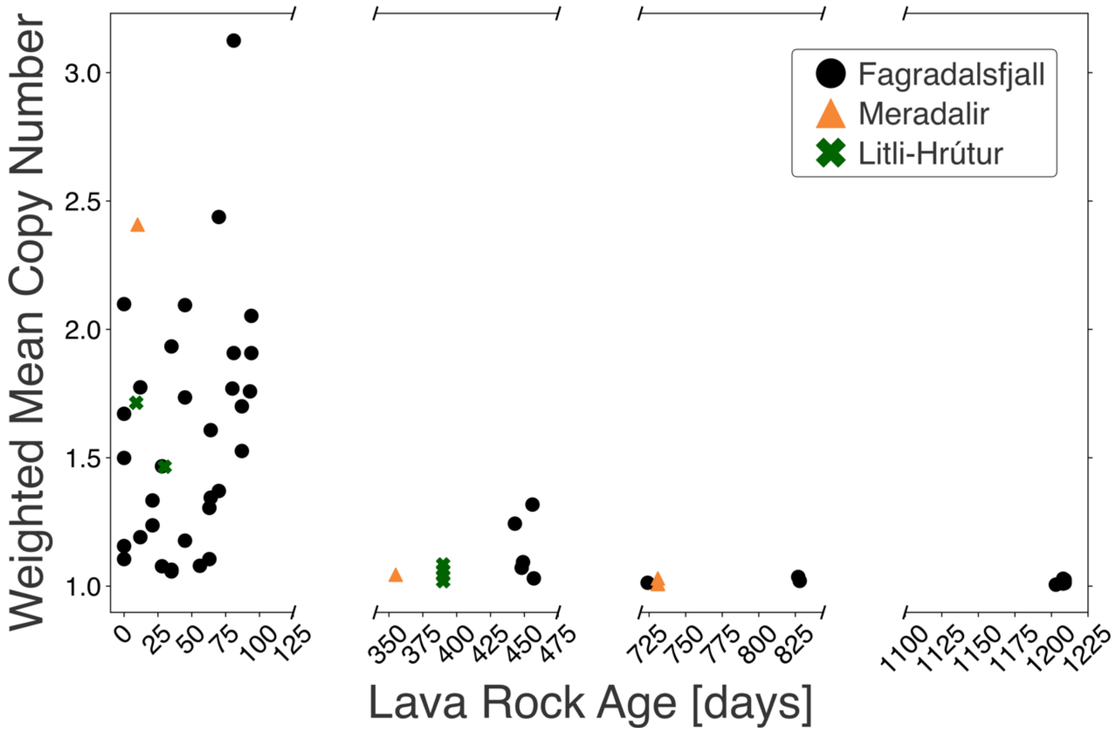

Supplementary Fig. 9. **Weighted mean 16S rRNA gene copy number vs lava rock age.** Data is separated with different markers for the three eruptions on a broken axis.

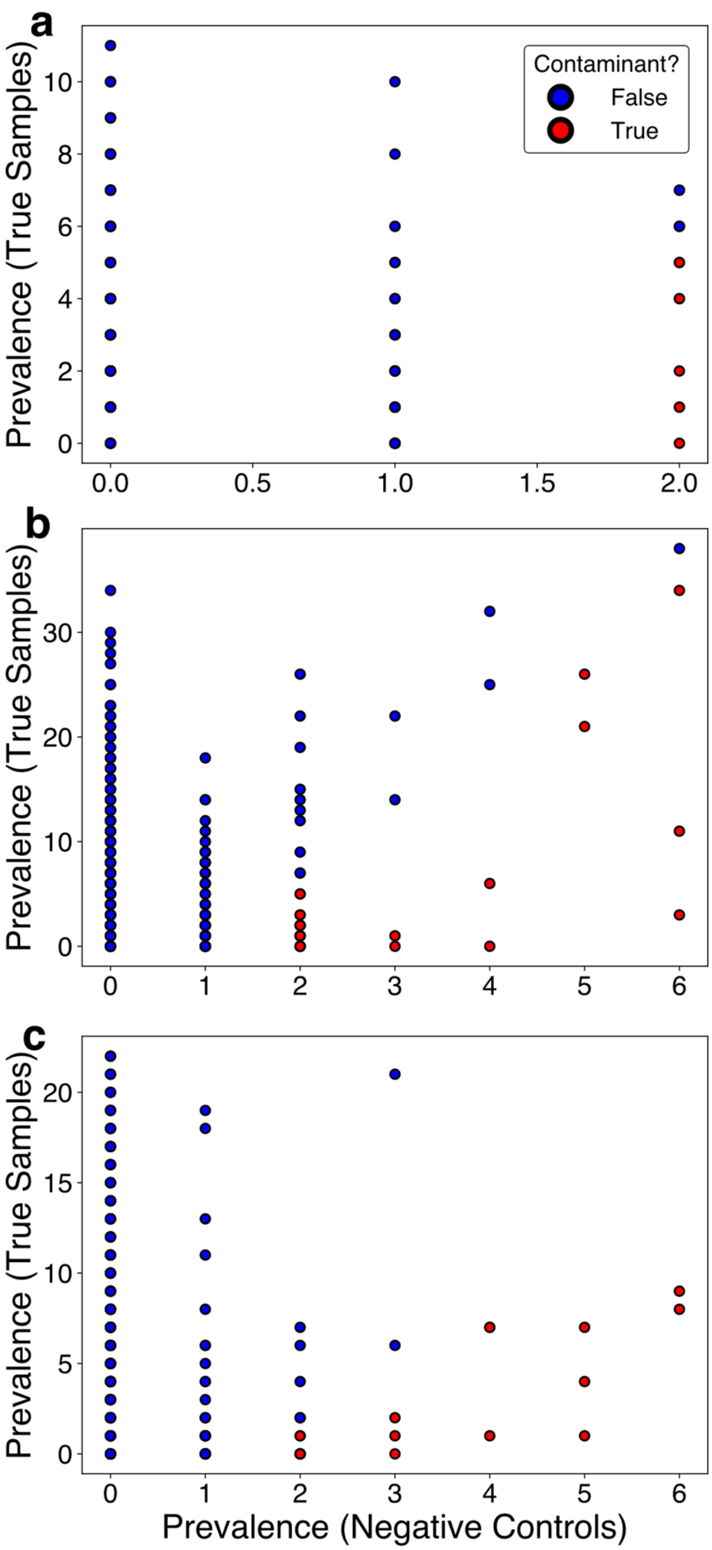

Supplementary Fig. 10. **Inferred contaminants using the prevalence method in decontam**. Different sequencing runs are shown separately (a–c) for lava extractions. A higher prevalence in the negative controls ($p < 0.1$) results in the sequence being identified as a contaminant.

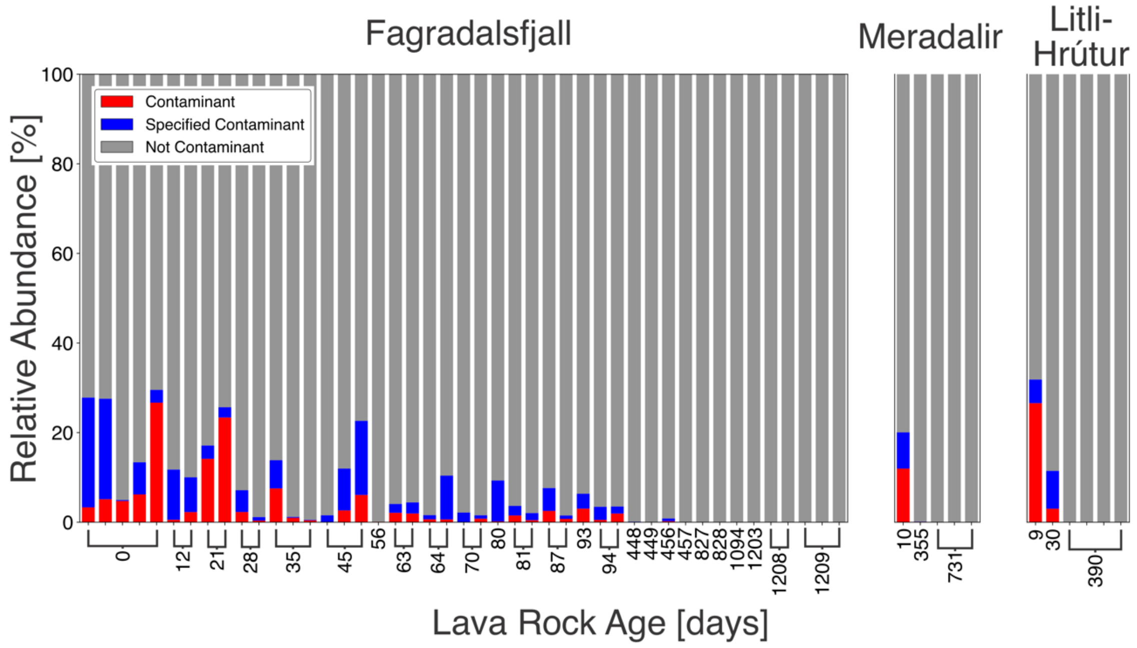

Supplementary Fig. 11. **Proportion of contaminants.** Due to the extraordinary nature of our samples and limited biomass, we chose to be rigorous with our treatment of our microbiome data. Contamination is a persistent problem in low biomass systems regardless of the methodology and therefore must be accounted for in downstream analyses. Contamination can be introduced during sampling, from reagents during sample processing, or from cross contamination with other samples. However, contaminants can be difficult to untangle from true signals due to tag-switching and cross-talk. While we employed rigorous efforts to mitigate contamination (see Main Text, Methods), potential contaminants, such as known skin microorganisms, were identified and removed (shown as “Specified Contaminants” in the plot). Additional decontamination was performed using the package decontam (see Supplementary Fig. 9) and are labelled as “Contaminant” on the plot.

Supplementary Table 1. Lava sample X-Ray Diffraction (XRD) results. Age values are in days unless otherwise specified (for old lava).

ID	Site Name	Age	Pyroxenes	Feldspars	Olivine	Magnesite	Unknown Peaks
L001	Gelingadalir	0	53%	42%	5%	0%	0%
L301	Litli-Hrútur Fissure	9	9%	82%	3%	0%	6%
L225	Meradalir Fissure	10	19.5%	41.5%	25%	5.5%	8.5%
L052	Site C	94	60%	33%	7%	0%	0%
L205	Site C	449	53.5%	41%	5.5%	0%	0%
L206	Shelly Site C	443	49%	48.%	2.5%	0%	0%
L209	Fumarole Site 1	345	56%	35.5%	8.5%	0%	0%
Ol301	12k-year old shield	12k years	38%	53.5%	8.5%	0%	0%
OL302	8k-year old flow	8k years	28%	69%	3%	0%	0%
OL303	2k-year old flow	2k years	42%	58%	0%	0%	0%

Supplementary Table 2. Cell counts vs environmental parameters: Linear fits excluding fumarole and old lava samples.

Parameter	Slope	Intercept	R^2
Age	182	9370	0.658
pH	3595	30141	0.003
Lava Rock Temperature	2251	24236	0.035
Water Content (%)	-4747	64455	0.082
Air Temperature	5792	-13035	0.102
Relative Humidity	593	11513	0.043
Wind Speed	-5736	86126	0.063

Supplementary Table 3. Successful cultures from lava and soil samples.

Sample Type	Site Name	Lava Age [days]	Isolated strain (% identity)	Media	Temperature [°C]
Lava	Site 2	56	*Sphingomonas echinoides* (95.2%)	R2A	50
Lava	Site 2	56	*Sphingomonas echinoides* (95.68%)	R2A	11
Lava	Site A	81	*Brevibacillus borstelensis* (95.45%)	R2A	50
Lava	Site A	94	*Bacillus licheniformis* (96.60%)	TSB	50
Lava	Site A	94	*Paenibacillus sp.* (97.27%)	TSB	11
Lava	Site A	94	*Bacillus licheniformis* (95.19%)	R2A	50
Lava	Fumarole	345	Not sequenced	BG-11	50
Lava	Fumarole	345	Not sequenced	R2A	11
Lava	Site B	448	Not sequenced	SOX	11
Soil	Site 3	N/A	*Bacillus licheniformis* (95.95%)	TSB	50
Soil	Site 3	N/A	*Pseudomonas sp.* (96.95%)	TSB	11

Supplementary Table 4. Linear mixed effects modeling results from relative abundances of taxa, Faith's Phylogenetic Diversity (PD) net relatedness index (NRI) and nearest taxon index (NTI). Percent variance due to site effects was calculated using intraclass correlation coefficient (ICC). Certain taxa varied in the first year of the study and therefore were subject to regression analyses <100 days. Estimated slopes are %/day or the alpha diversity index per day.

Parameter	Slope [value/day]	Standard Error	*p*-value	Variance from ICC for site	Regression <100 days
Udaeobacter	0.259	0.114	0.031	40.8%	Y
Micrococcales	-0.008	0.002	0.000	21.0%	N
Acidiphilium	0.024	0.002	0.000	3.4%	N
Beijerinckiaceae	0.018	0.005	0.001	0.0%	N
Granulicella	0.009	0.001	0.000	0.0%	N
NRI	-0.003	0.001	0.000	0.7%	N
NRI	0.006	0.010	0.571	1.9%	Y
NTI	-0.002	0.001	0.000	23.0%	N
NTI	0.01	0.009	0.270	30.0%	Y
Faith's PD	-0.002	0.005	0.755	26.1%	N
Faith's PD	0.259	0.114	0.031	40.8%	Y
