# Supplementary material for: Three eruptions at the Fagradalsfjall Volcano in Iceland show rapid and predictable microbial community establishment
Source: Commun Biol. 2025 Nov 24;8:1657. doi: 10.1038/s42003-025-09044-1 (PMC12644735; doi:10.1038/s42003-025-09044-1)
Supplement: Supplementary file 3 — Description of Additional Supplementary files [file 42003_2025_9044_MOESM3_ESM.pdf]

Description of Additional Supplementary files

File name: Supplementary_Data1.xlsx

Description: Lava sample list and environmental parameters

File name: Supplementary_Data2.xlsx

Description: The source data behind the graphs in the paper
