# Supplementary material for: Three eruptions at the Fagradalsfjall Volcano in Iceland show rapid and predictable microbial community establishment
Source: Commun Biol. 2025 Nov 24;8:1657. doi: 10.1038/s42003-025-09044-1 (PMC12644735; doi:10.1038/s42003-025-09044-1)
Supplement: Supplementary file 6 — Reporting Summary [file 42003_2025_9044_MOESM6_ESM.pdf]

nature portfolio

Corresponding author(s): Solange Duhamel

Last updated by author(s): Aug 13, 2025

Reporting Summary

Nature Portfolio wishes to improve the reproducibility of the work that we publish. This form provides structure for consistency and transparency in reporting. For further information on Nature Portfolio policies, see our Editorial Policies and the Editorial Policy Checklist.

Statistics

For all statistical analyses, confirm that the following items are present in the figure legend, table legend, main text, or Methods section.

n/a	Confirmed	
☐	☒	The exact sample size (*n*) for each experimental group/condition, given as a discrete number and unit of measurement
☐	☒	A statement on whether measurements were taken from distinct samples or whether the same sample was measured repeatedly
☐	☒	The statistical test(s) used AND whether they are one- or two-sided<br>*Only common tests should be described solely by name; describe more complex techniques in the Methods section.*
☐	☒	A description of all covariates tested
☐	☒	A description of any assumptions or corrections, such as tests of normality and adjustment for multiple comparisons
☐	☒	A full description of the statistical parameters including central tendency (e.g. means) or other basic estimates (e.g. regression coefficient) AND variation (e.g. standard deviation) or associated estimates of uncertainty (e.g. confidence intervals)
☐	☒	For null hypothesis testing, the test statistic (e.g. *F*, *t*, *r*) with confidence intervals, effect sizes, degrees of freedom and *P* value noted<br>*Give P values as exact values whenever suitable.*
☐	☒	For Bayesian analysis, information on the choice of priors and Markov chain Monte Carlo settings
☒	☐	For hierarchical and complex designs, identification of the appropriate level for tests and full reporting of outcomes
☐	☒	Estimates of effect sizes (e.g. Cohen's *d*, Pearson's *r*), indicating how they were calculated

Our web collection on statistics for biologists contains articles on many of the points above.

Software and code

Policy information about availability of computer code

Data collection	No software was used for data collection.
Data analysis	All data was analyzed in Python. Sequencing data was processed using QIIME2, DADA2, and decontam. Statistics were conducted using QIIME2. Bayesian source tracking was conducted using SourceTracker2. Phylogenetic null modelling was conducted using the microeco R package. Linear Mixed Effects models were analyzed using pymer4. Random forest modelling was conducted using QIIME2's sample classifier plugin. Metabolic inference was conducted using paprica, FAPROTAX, and PICRUSt2. No custom software was developed.

For manuscripts utilizing custom algorithms or software that are central to the research but not yet described in published literature, software must be made available to editors and reviewers. We strongly encourage code deposition in a community repository (e.g. GitHub). See the Nature Portfolio guidelines for submitting code & software for further information.

Data

Policy information about availability of data

All manuscripts must include a data availability statement. This statement should provide the following information, where applicable:

- Accession codes, unique identifiers, or web links for publicly available datasets
- A description of any restrictions on data availability
- For clinical datasets or third party data, please ensure that the statement adheres to our policy

Raw sequences are available in the GenBank Sequence Read Archive under accession numbers SAMN46055121–SAMN46055246, BioProject accession number

PRJNA1205709. GenBank accession numbers for the strains isolated during this work are PQ835131–PQ835142. Environmental covariates are available in Supplementary Tables 1 and 2. Data for plots presented in the main manuscript are available in the Supplementary Data. Samples are available upon reasonable request.

Research involving human participants, their data, or biological material

Policy information about studies with human participants or human data. See also policy information about sex, gender (identity/presentation), and sexual orientation and race, ethnicity and racism.

Reporting on sex and gender	N/A
Reporting on race, ethnicity, or other socially relevant groupings	N/A
Population characteristics	N/A
Recruitment	N/A
Ethics oversight	N/A

Note that full information on the approval of the study protocol must also be provided in the manuscript.

Field-specific reporting

Please select the one below that is the best fit for your research. If you are not sure, read the appropriate sections before making your selection.

☐ Life sciences ☐ Behavioural & social sciences ☒ Ecological, evolutionary & environmental sciences

For a reference copy of the document with all sections, see nature.com/documents/nr-reporting-summary-flat.pdf

Ecological, evolutionary & environmental sciences study design

All studies must disclose on these points even when the disclosure is negative.

Study description	This study analyzes the transition from uninhabited to inhabited natural substrates by investigating a series of effusive volcanic eruptions in Iceland. A series of fixed sites were established as the eruptions progressed and lava was sampled over the course of three years to document the development of the microbial community.
Research sample	Freshly emplaced lava rocks and potential source environments (soil, aerosol, hot spring, rainwater, old lava).
Sampling strategy	Pieces of lava were aseptically broken off the lava flow using an ethanol and flame-sterilized rock hammer and collected into a double-bagged sterile Whirl-Pak. Sterile gloves and facemasks were also used during sampling. Bioaerosol samples were collected via impaction onto quartz membrane filters (Pall Corporation) using the Deployable Particulate Sampler (SKC, Inc.) with a PM10 selective inlet or a SASS 3100 instrument mounted 1.5 m above the ground. Rain samples were collected using a sterile plastic cup and then filtered onto Sterivex. Hot spring samples were similarly filtered onto Sterivex. Soil samples were collected aseptically by scraping the surface into 15 mL polypropylene tubes using an ethanol and flame sterilized spoon and then double bagged in sterile Whirlpaks for storage. Sample sizes were chosen to obtain sufficient material for downstream bulk analyses (e.g., filling Whirlpaks with lava).
Data collection	Field data (e.g., ambient and lava temperature with a K-type thermocouple, pH, contextual imaging of sampling sites using DJI Mavic series drones) were collected by all team members throughout the duration of the field campaigns. Weather data for multivariate analyses of the microbiome data was obtained from the Festerfjall station (63.85948°N, 22.34358°W) from the Icelandic Meteorological Office. XRD data was collected with the Philips PANalytical X'Pert PRO MPD instrument. Substrate utilization profiling was performed with an EcoPlate and well color development analyzed using a SpectraMax iD5 plate reader (Molecular Devices). Cell counts were conducted using a 60× magnification on an epifluorescence microscope (Nikon) at the University of Arizona. Culture DNA was extracted using the PureLink Microbiome DNA Extraction Kit and analyzed for Sanger sequencing on an Applied Biosystems 3730XL DNA Analyzer. DNA from environmental samples was extracted using commercial kits depending on the sample type and with special modifications for low biomass samples. Sequencing was performed at MR DNA (Shallowater, TX, USA) on a MiSeq.
Timing and spatial scale	Sampling and data collection began on March 20th, 2021 and ended on August 6th, 2024. Frequency of sampling was limited by logistic feasibility, weather conditions, and eruption safety. For example, members of our team were on a Fulbright fellowship for the 6-month duration of the 2021 eruption, enabling weekly to biweekly sampling in order to provide high resolution development of the microbial community. Sampling in subsequent years was limited to a few field days due to logistical and financial constraints and only in the summer due to more mild weather conditions during those months. We were fortuitously present for the 2022 and 2023 eruptions which enabled sampling of additional extremely young lava. Sites were strategically established to prevent overprinting by subsequent lava flows and as distant as possible from tourist access trails while retaining accessibility for frequent sampling trips. Consequently, sites were established mostly within the same areas for the respective eruption due to the limited areas that met these criteria.

Data exclusions	One sample, lava sample L203 (see Supplementary Table 2), had poor sequencing data quality and therefore was re-sequenced in a subsequent run. Therefore, the original data was excluded from data analysis.
Reproducibility	To verify the reproducibility of our findings, we leveraged both spatial and temporal replication. Multiple sites were established within the same lava flow to verify trends in microbial community development. Moreover, as the same volcanic system erupted in 2021, 2022 and 2023, we were able to monitor a natural ecological triplicate. This kind of replication is extremely rare in environmental sciences and therefore makes our conclusions robust. Additional replication was conducted in attempts at culturing lava and cell counts (e.g., two slides were prepared per sample). Substrate utilization assays have replicates built into the 96 well plates.
Randomization	Samples were grouped by eruption. Samples from the surface of the lava flows were randomly collected.
Blinding	Given the logistical and environmental constraints of the fieldwork, formal blinding was not employed during data acquisition. However, sample collection followed standardized protocols across all sites and years to minimize subjective bias. Sample processing and sequencing were performed using consistent laboratory procedures, with negative controls and blanks included at each step to monitor for contamination. However, to minimize bias during downstream analysis, all sequencing data were processed using standardized, previously published pipelines in QIIME2. Analyses of diversity and community composition were performed using script-based workflows without prior knowledge of expected outcomes, helping to reduce subjective interpretation.

Did the study involve field work? ☒ Yes ☐ No

Field work, collection and transport

Field conditions	Iceland has a temperate subarctic climate. Field work took place over the course of three years and in different seasons. Environmental conditions at the time of sample collection for each lava sample is available in Supplementary Table 2.
Location	Sampling was conducted at a variety of locations at the Fagradalsfjall volcano on the Reykjanes peninsula in southwestern Iceland. A full list of sampling site locations is available in Supplementary Table 2. The main sampling area at Fagradalsfjall was located around 63.8952 N 22.2603 W at 205 m elevation. For the 2022 Meradalir eruption, the main site was at 63.9001 N 22.2470 W at 175 m. The main site for Litli Hrutur was at 63.9219 N 22.2006 W at 194 m.
Access & import/export	Access to the field site was obtained in collaboration with the University of Iceland and The Icelandic Association for Search and Rescue. All sampling at Fagradalsfjall and sample export was done under permits from the Icelandic Institute of Natural History, which was valid April 29th, 2021 to August 31st, 2024.
Disturbance	All site access followed designated 4x4 vehicle routes established by the Icelandic Search and Rescue team specifically for scientific purposes, ensuring minimal disturbance to the surrounding landscape. Lava samples were collected several meters inward from the flow margin to avoid visible disruption along the edges of the flow field. Collection was performed using aseptic techniques and targeted fragments in a way that preserved the natural appearance of the site. No markers, excavation, or other lasting alterations were introduced, ensuring that sampling left no noticeable trace.

Reporting for specific materials, systems and methods

We require information from authors about some types of materials, experimental systems and methods used in many studies. Here, indicate whether each material, system or method listed is relevant to your study. If you are not sure if a list item applies to your research, read the appropriate section before selecting a response.

Materials & experimental systems

n/a	Involved in the study
☒	☐ Antibodies
☒	☐ Eukaryotic cell lines
☒	☐ Palaeontology and archaeology
☒	☐ Animals and other organisms
☒	☐ Clinical data
☒	☐ Dual use research of concern
☒	☐ Plants

Methods

n/a	Involved in the study
☒	☐ ChIP-seq
☒	☐ Flow cytometry
☒	☐ MRI-based neuroimaging

Plants

Seed stocks	*Report on the source of all seed stocks or other plant material used. If applicable, state the seed stock centre and catalogue number. If plant specimens were collected from the field, describe the collection location, date and sampling procedures.*
Novel plant genotypes	*Describe the methods by which all novel plant genotypes were produced. This includes those generated by transgenic approaches, gene editing, chemical/radiation-based mutagenesis and hybridization. For transgenic lines, describe the transformation method, the number of independent lines analyzed and the generation upon which experiments were performed. For gene-edited lines, describe the editor used, the endogenous sequence targeted for editing, the targeting guide RNA sequence (if applicable) and how the editor was applied.*
Authentication	*Describe any authentication procedures for each seed stock used or novel genotype generated. Describe any experiments used to assess the effect of a mutation and, where applicable, how potential secondary effects (e.g. second site T-DNA insertions, mosiacism, off-target gene editing) were examined.*
